# Duration of fixed appliance treatment using 0.018-inch slot versus 0.022-inch slot brackets: a systematic review and meta-analysis

**DOI:** 10.1093/ejo/cjaf082

**Published:** 2025-10-14

**Authors:** Mumen Z Rizk, Hisham Mohammed, John Samy, Grant McIntyre, David Bearn

**Affiliations:** Private Practice, Cairo, Egypt; School of Dentistry, University of Queensland, QLD 4006, Australia; Private Practice, Cairo, Egypt; School of Dentistry, University of Dundee, Dundee DD1 4HR, United Kingdom; School of Dentistry, University of Dundee, Dundee DD1 4HR, United Kingdom

**Keywords:** fixed appliances, orthodontic treatment duration, bracket slot size

## Abstract

**Background:**

The duration of fixed orthodontic appliance treatment is a major concern for both patients and clinicians. A lengthy treatment increases the likelihood of iatrogenic effects and burdens patient compliance.

**Objectives:**

The primary aim of this review is to compare the duration of orthodontic treatment between 0.018-inch and 0.022-inch slot brackets. Furthermore, the quality of treatment, iatrogenic effects, and patient perception will be explored as secondary outcomes.

**Search methods:**

An unrestricted comprehensive search was undertaken on six electronic databases updated up to May 2025. Moreover, ongoing and unpublished studies were searched on relevant sources. The reference lists of retrieved studies were screened for potential studies.

**Selection criteria:**

Only randomized clinical trials (RCTs) and prospective non-randomized studies (NRS) with parallel-arm design were selected for inclusion.

**Data collection and analysis:**

Study selection, bias assessment, and data extraction were performed independently by two reviewers, and inconsistencies were resolved by a third reviewer. Quantitative analyses were undertaken for comparable studies presented as a random-effects model along with its 95% confidence intervals (CI). Additional sensitivity and subgroup analyses were performed. The generated summary effect was evaluated using the GRADE approach.

**Results:**

Twelve articles involving nine unique studies (six RCTs and three NRS: 832 patients) met the inclusion criteria. The total duration of treatment did not differ between the bracket slot systems (standardized mean difference: −0.23; 95% CI: −0.68 to 0.23; I^2^: 24%; 4 studies; low level of evidence). Qualitative analysis revealed no significant differences between the 0.018-inch and 0.022-inch slot brackets regarding the quality of treatment outcomes, iatrogenic treatment effects, or patient-related outcomes.

**Limitations:**

Differences in reporting and clinical heterogeneity precluded meta-analysis for the secondary review outcomes.

**Conclusions and implications:**

Within the limitations of this systematic review, there is some low-quality evidence suggesting that bracket slot size does not significantly impact orthodontic treatment in terms of duration, treatment outcomes, or patient-centred variables.

**Registration:**

PROSPERO CRD42019121569.

## Introduction

Fixed appliances are the most widely used modality for orthodontic treatment. Since the standard edgewise appliance was introduced in the late 1920s, multiple modifications have been made to the original design. Pre-adjusted edgewise orthodontic appliances are manufactured in various materials, ligation methods, and slot sizes. Contemporary fixed appliances are largely available in the original 0.022 × 0.028-inch slot and 0.018 × 0.022-inch slot sizes. This variation in bracket systems has sparked debate within the orthodontic community, particularly regarding the effectiveness and efficiency of bracket slot sizes and ligation methods.

Clinicians claim different advantages for the use of 0.018-inch and 0.022-inch bracket slots. Some advocate for the 0.022-inch slot as it allows for a generous clearance during the alignment stage and stiffer archwires during space closure, offering lighter forces and better control. Others claim that the 0.018-inch slot facilitates quicker archwire progression, leading to an early expression of the built-in prescription, particularly torque in the anterior segment [1]. These claims have sparked an ongoing debate among clinicians regarding bracket slot size selection, which might complicate the transfer of orthodontic patients [2]. Moreover, this lack of consensus has led to multiple attempts to combine the perceived benefits of both systems, including the promotion of a standardized 0.020-inch bracket slot [3, 4] and a bi-dimensional technique [5].

Orthodontic fixed appliance treatment can typically last up to 20 months [6]. The duration of orthodontic treatment seems to significantly affect the general attitude towards orthodontics in both treated and untreated populations [7–9]. Furthermore, prolonged orthodontic treatment adversely affect patients’ compliance [10], exacerbating iatrogenic effects such as root resorption, demineralization, gingival, and periodontal damage [11] and is related to discontinuation of treatment [12]. Predictable short treatment duration is linked to increased patient satisfaction [13] and cost-effectiveness [14].

Much of the literature comparing 0.018-inch and 0.022-inch bracket slots has relied on retrospective data and therefore offered inconclusive results [15]. However, recent primary research publications have added to our knowledge on this topic. Currently, there is still a lack of consensus amongst clinicians on which bracket slot size to use during fixed appliance treatment. This review aims to compare the duration of orthodontic treatment between 0.018-inch and 0.022-inch slot brackets during different stages of orthodontic treatment. Moreover, the effect of the bracket slot size on the quality of treatment, iatrogenic effects, and patient perception will be explored.

## Materials and methods

### Protocol registration

The protocol for this review was registered *a priori* in the International Prospective Register of Systematic Reviews PROSPERO (CRD42019121569). This review was planned and reported in accordance with the recommendations outlined by the preferred reporting items for systematic reviews and meta-analyses (PRISMA) [16] and the Cochrane Handbook for Systematic Reviews of Interventions [17].

### Eligibility criteria for included studies

#### Population

Orthodontic patients of any age with fixed appliance treatment.

#### Intervention

Orthodontic fixed appliance treatment using 0.018-inch slot brackets.

#### Comparison

Orthodontic fixed appliance treatment using 0.022-inch slot brackets.

#### Outcomes

Main outcome:

- Duration of treatment

Additional outcomes:

- Iatrogenic effects of treatment
- Quality of orthodontic treatment outcome
- Patient-related outcomes (pain and oral health-related quality of life (OHRQoL))
- Cost-effectiveness (including direct, indirect, and intangible costs)

#### Type of included studies

Randomized clinical trials (RCTs) and prospective non-randomized studies (NRS) of parallel design. There were no limitations on language or date of study publication. *Ex vivo* studies, non-human studies, case reports, review articles, and retrospective studies were excluded.

### Information sources, search, and study selection

A combination of keyword terms and medical subject headings was used to search six electronic databases (Cochrane central register of controlled trials, MEDLINE, PubMed, Embase, Scopus, and Web of Science) updated up to May 2025 with no restrictions on language, publication date or study design. Four additional databases were searched for relevant ongoing and unpublished trials (Supplementary Table S1). Moreover, a manual search targeting the reference lists of potentially included studies and relevant review articles was also conducted.

Search and study selection were carried out by two independent reviewers (M.R. and H.M.) with a third reviewer (J.S.) acting as a mediator. Titles and abstracts of retrieved studies were assessed against the inclusion and exclusion criteria, followed by a full-text eligibility assessment. Authors were to be contacted to enquire about missing data and for clarification, if required. Studies excluded based on their full text were recorded along with the reasons for their exclusion.

### Data collection

Data collection was performed in duplicate by two independent reviewers (M.R. and H.M.) using a pre-piloted customized data collection form. Collected data items were related to study identification, study design, study setting, sample size, along with the participant’s age and gender, specification of brackets utilized, and relevant reported outcomes. Conflicts were mediated by a third reviewer (J.S.).

### Risk of bias in individual studies

The Cochrane risk of bias tool (RoB 2.0) [18] for RCTs was utilized to assess bias arising from the randomization process, bias due to deviations from the intended interventions, missing outcome data, the measurement of outcomes, and the selection process of the reported result. Bias introduced by the NRS was assessed using ROBINS-I tool [19] based on seven different domains, including confounding bias, selection bias, classification of intervention, deviations from interventions, missing data, measurement of outcomes, and selection of results. The assessment was carried out independently by two reviewers (M.R. and H.M.), and a third reviewer (J.S.) mediated disagreement.

### Summary measures and approach to data synthesis

A structured qualitative narrative synthesis was provided. For continuous data, the mean scores alongside their standard deviations were pooled together and summarized as standardized mean difference (SMD) with its corresponding 95% confidence interval (CI) [20]. The choice of SMD was deemed appropriate to accommodate different time scales (i.e. years versus months), in accordance with the recommendations outlined within the Cochrane Handbook for Systematic reviews. A random-effects model was used as per DerSimonian and Laird [21] with weightings adjusting for potential heterogeneity, bias, and confounding, and appropriated with the Knapp–Hartung adjustment. Clinical heterogeneity was accounted for by assessing inter-study variances with particular attention to measurement methods, appliance type, ligation method, and intervals of data collection. Statistical heterogeneity was gauged via graphical inspection of displayed estimated effects along with their 95% CIs. Furthermore, the I^2^ and tau^2^ statistics were utilized to quantify statistical heterogeneity. When three or more studies were meta-analysed, the 95% prediction interval (Prl) was calculated to display plausible future observations. The GRADE approach was used to summarize the resultant cumulative evidence from the meta-analysis.

### Risk of bias across studies and additional analyses

Publication bias and ‘small study’ effects were planned to be inspected through the graphical examination of the generated funnel plots and calculation of Egger’s linear regression with meta-analyses involving 10 or more studies. In addition, pertinent subgroup and sensitivity analyses were planned to identify and isolate the effects of study design and methodological quality on the summary effect measures.

## Results

### Study selection

The initial electronic and manual searches yielded a total of 680 references. After duplicates were removed using EndNote reference management software (Clarivate Analytics, Philadelphia, Pennsylvania, USA), the remaining 226 references were screened for relevance based on their title and abstracts. The full text of 21 articles was retrieved and reviewed, and nine articles were excluded as they failed to meet the inclusion criteria (Supplementary Table S2).

**Figure 1.**
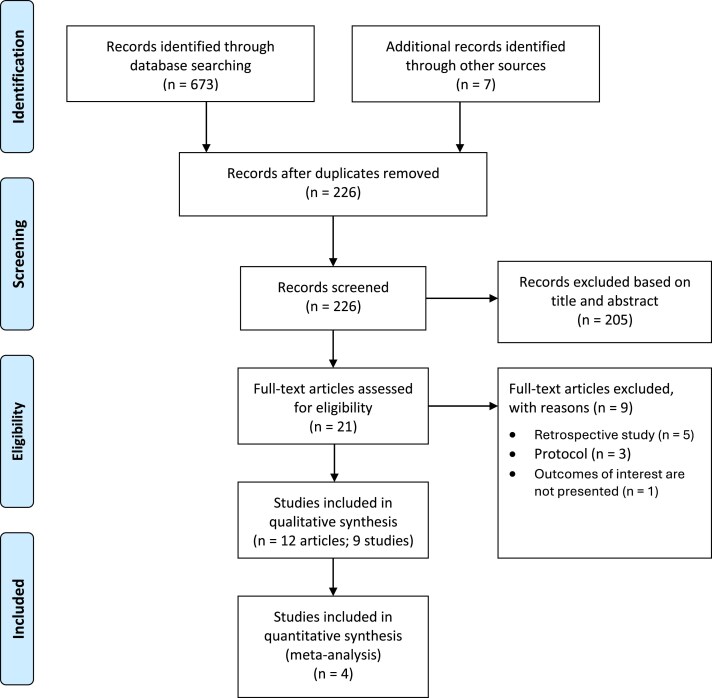
PRISMA flow diagram.

Twelve articles (nine studies) fulfilled the inclusion criteria, including a final sample of six RCTs [22–29] and three NRS [30–32]. The PRISMA flow chart presents the selection process (Fig. 1).

### Characteristics of the participants

The nine included studies approximately involved a total of 832 participants aged 10 to 50 years. Studies were undertaken in nine different countries; one large RCT published in four parts was undertaken in the UK [27–29], while the others took place in the Netherlands, Kuwait, USA, Egypt, India, Spain, Australia, and Iran. All but two studies [30, 31] were single-centre investigations. Two articles [28, 30] focused on comparing the duration of treatment between the two systems. Treatment duration was also reported in two other articles [26, 32], though they primarily focused on biological side effects, which were also explored in two additional publications [27, 31]. Two articles [23, 28] reported on the quality of treatment outcomes, while four studies investigated alignment efficiency and duration [22–24, 28]. Patient-related outcomes were reported by two of the included studies [25, 28]. Studies included a range of malocclusions, primarily Class I and Class II. Most studies had similar exclusion criteria, excluding participants with a history of previous orthodontic treatment, functional appliances and those requiring extractions other than premolars or combined orthodontic-orthognathic surgery. Furthermore, these studies attempted to control for confounding factors such as the amount of tooth displacement, irregularity index (II), extraction pattern, history of trauma, or periodontal disease. Three studies [22, 25, 32] adopted a non-extraction approach, while the others included a mix of extraction and non-extraction cases. Extraction spaces were mainly closed using sliding mechanics, with intermaxillary elastics employed as necessary. Despite a wide age range within the samples, the majority of studies focused on fixed appliance treatment, employed a standard protocol of care, used similar treatment techniques in both groups and excluded participants who had undergone previous functional appliance treatment. A detailed description of the characteristics of the included studies is provided in Table 1.

**Table 1.** Characteristics of the included studies.

Study	Design	Setting	Participants	Malocclusion	Bracket type	Outcomes	Conclusions
Abouwafia *et al.* [22]	RCT	Future University, Egypt	G1 (0.018″) *n* = 7 2 (0.022″) *n* = 7	Mild to moderate crowding Normal overjet and overbite (2–4 mm)	Edgewise Mini (Roth)	Alignment duration	The 0.018-inch group achieved alignment 1 month faster than the 0.022-inch group
Amditis and Smith [30]	NRS (pCCT)	Multicentre private practice, Australia	G1 (0.018″) *n* = 32 (12 M and 21 F) Mean age 15.6 y G2 (0.022″) *n* = 32 (9 M and 23 F) Mean age 14.9 y	Class I, Class II div 1, Class II div 2 and Class III Mean mandibular plane angle (33.4°) Mean overjet (3.1 mm) and mean overbite (2.5–2.7 mm)	Edgewise (Roth)	Overall TTT duration Number of appointments	The overall TTT duration was one month less in the 0.018-inch group The 0.018-inch group had fewer appointments
Artun *et al.* [31]	NRS (pCCT)	Multicentre in Kuwait, Nijmegen, The Netherlands, and Seattle, Wash.	G1 (0.018″) *n* = 113 G2 (0.022″) *n* = 189 Mean age 18.8 y	Various malocclusions	Multi-bonded pre-adjusted appliances	Iatrogenic effects (OIIRR)	The bracket slot size did not affect the severity of OIIRR
Bhardwaj *et al.* [23]	RCT	Institute of Dental Studies and Technologies, India	G1 (0.018″) *n* = 14 (Self‑ligating *n* = 7, self‑ligating tandem *n* = 7) G2 (0.022″) *n* = 21 (Conventional *n* = 7, self‑ligating *n* = 7, self‑ligating tandem *n* = 7) Age (14–25) y	Class I and Class II div 1 (6–15 mm) incisor irregularity index (II)	Edgewise conventional (MBT) and self-ligating	Quality of treatment	No statistically significant differences between the two groups
Cobb *et al.* [24]	RCT	University of North Carolina, USA	G1 (0.018″) *n* = 85 arches G2 (0.022″) *n* = 70 arches	Various malocclusions No anterior vertical misposition > 3.0 mm or completely blocked out II > 5.0 mm	Edgewise (mix of single wing and twin brackets)	Alignment duration	No difference in the maxilla, however, alignment was faster in the mandibular arch for the 0.022-inch group
Curto *et al.* [25]	RCT	University of Salamanca, Spain	G1 conventional (0.018″) *n* = 30 (17 M and 13 F) Mean age 21.7 y G2 conventional (0.022″) *n* = 30 (15 M and 15 F) Mean age 23.5 y G3 low-friction (0.018″) *n* = 30 (15 M and 15 F) Mean age 24 y G4 low-friction (0.022″) *n* = 30 (14 M and 16 F) Mean age 22.6 y	Crowding between 1.0 and 2.0 mm in both arches Skeletal Class I or Class II	Edgewise conventional and low-friction	Patient-related outcomes (Physical pain and Oral Health Impact Profile)	The 0.022-inch group had less pain and a better OHRQoL
Reukers *et al.* [26]	RCT	University of Nijmegen, the Netherlands	G1 partly programmed (0.018″) G2 fully programmed (0.022″) *n* = 149 (64 M and 85 F). Mean age 12 y 4 mo	Class II malocclusion	Fully programmed edgewise appliance (Roth) and a partly programmed edgewise appliance (Microloc)	Overall TTT duration Iatrogenic effects (OIIRR)	No significant differences between the groups The bracket slot size did not affect the severity of OIIRR
Sobouti *et al.* [32]	NRS (pCCT)	Mazandaran University of Medical Sciences, Iran	G1 (0.018″) *n* = 360 teeth (7 M and 11 F) Mean age 16 y G2 (0.022″) *n* = 360 teeth (9 M and 9 F) Mean age 14 y	Class l crowding malocclusion	Edgewise	Overall TTT duration Iatrogenic effects (OIIRR)	The bracket slot size did not affect the severity of OIIRR
UK study [27–29]	RCT	University of Dundee, UK	G1 (0.018″) *n* = 77 (21 M and 56 F) Mean age 19.41 y G2 (0.022″) *n* = 76 (27 M and 49 F) Mean age 18.67 y	Various malocclusions	Edgewise (MBT)	Alignment duration Quality of treatment Overall TTT duration Number of appointments Iatrogenic effects (OIIRR) Patient-related outcomes	No significant difference in alignment duration No significant differences between the groups in the quality of the outcomes No significant differences in pain perception The bracket slot size did not affect the severity of OIIRR

F, female; G, group; II, irregularity index; M, male; mo, months; NRS, non-randomized study; OHRQoL, oral health-related quality of life; OIIRR, orthodontically induced inflammatory root resorption; pCCT, prospective controlled clinical trial; RCT, randomized clinical trial; TTT, treatment; y, year.

### Risk of bias in individual studies

**Figure 2.**
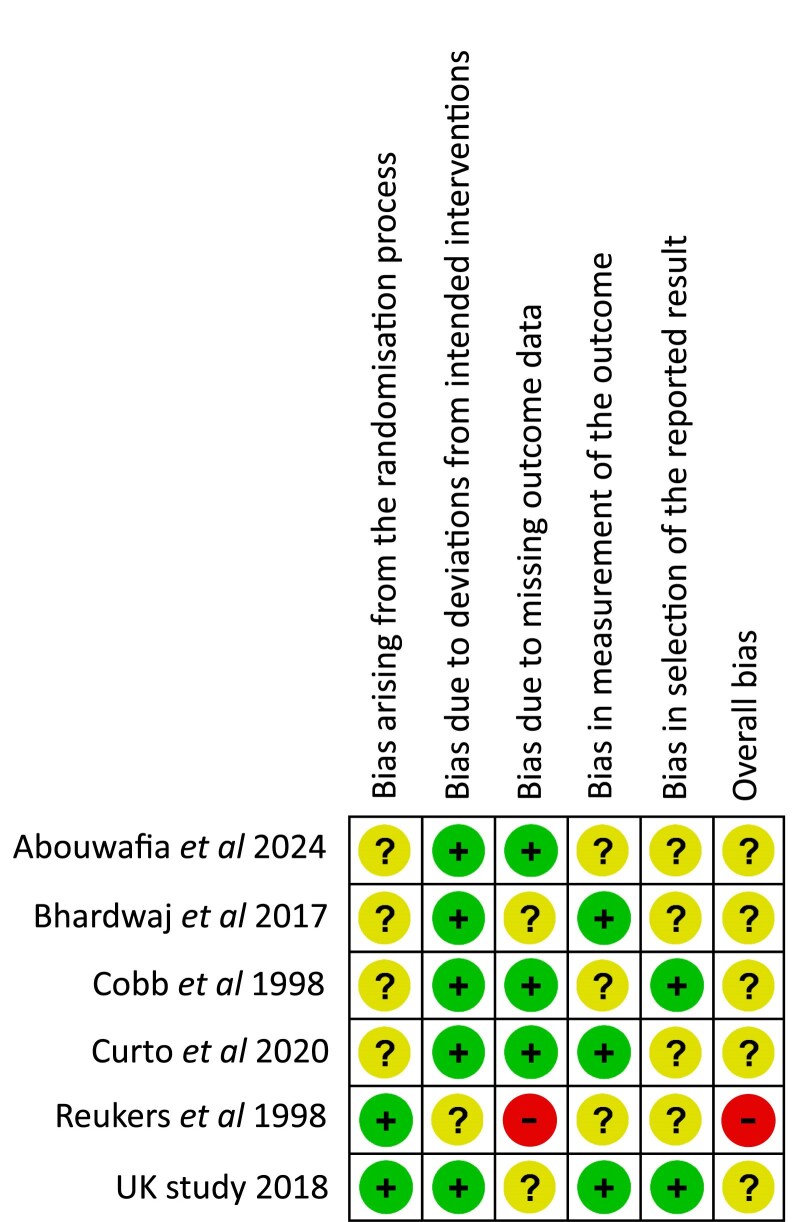
Risk of bias summary of the included RCTs.

**Figure 3.**
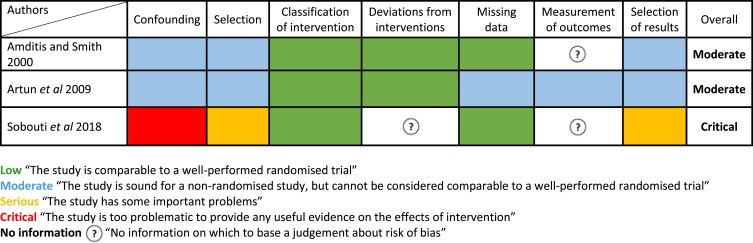
ROBINS-I tool for the risk of bias in the included NRS.

The ROB 2.0 tool revealed the risk of bias was unclear for five of the six included RCTs [22–25, 27–29], which was mainly related to participant dropout rates, introducing potential bias due to missing outcome data. Additionally, the risk of bias arising from the randomization process was unclear for four RCTs [22–25], and for the selection of reported results domain, a further four RCTs [22, 23, 25, 26], the risk of bias was unclear. One RCT scored a high risk of bias due to significant missing outcome data [26]. Risk of bias for the included RCTs is illustrated in Fig. 2. The risk of bias for two of the included NRS was moderate [30, 31], mainly due to confounding and selection bias, along with missing data and selective reporting of results. The remaining NRS was judged to have a critical risk of bias [32] (Fig. 3).

### Results of individual studies

#### Duration of treatment

**Figure 4.**
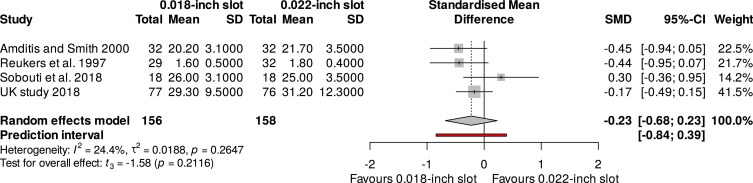
Meta-analysis of the overall treatment duration for the 0.018-inch and 0.022-inch slot systems.

**Figure 5.**
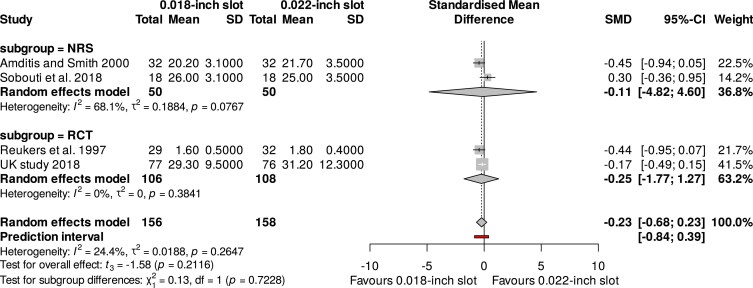
Subgroup analysis of the overall treatment duration between randomized and non-randomized studies.

An exploratory meta-analysis of four studies, including a total of 314 participants, revealed non-significant differences regarding the overall treatment duration for the 0.018-inch and 0.022-inch slot systems (SMD: −0.23; 95% CI: −0.68 to 0.23; I^2^: 24%; 95% Prl: −0.84 to 0.39; 4 studies) (Fig. 4). Subgroup analysis of RCTs and NRS did not have a significant impact on the outcomes of the meta-analysis (*P*-value = 0.72; Fig. 5). Again, sensitivity analysis by excluding high risk of bias studies [26, 32] did not impact the meta-analysis results (SMD: −0.25; 95% CI: −1.84 to 1.34; I^2^: 0%; Tau^2^: 0; 2 studies). The overall GRADE summary recommendation for the total duration of treatment with 0.018-inch and 0.022-inch slot sizes was low (Table 2).

**Table 2.** GRADE assessment of the quality of evidence for treatment duration.

Certainty assessment							No. of patients		Effect		Certainty	Importance
No. of studies	Study design	Risk of bias	Inconsistency	Indirectness	Imprecision	Other considerations	0.018-inch slot size bracket	0.022-inch slot size bracket	Relative (95% CI)	Absolute (95% CI)		
**Duration of treatment (assessed with: Standardized mean difference)**												
4	Randomized and non-randomized prospective studies	Very serious^a^	Not serious	Not serious	Not serious	None	156	158	—	(SMD **0.23 SD lower** in the 0.018-inch slot group (0.68 lower to 0.23 higher)	⨁⨁◯◯ Low^a^	Duration of orthodontic treatment is a critical domain from a healthcare and patients’ perspectives.

CI, confidence interval; SMD, standardized mean difference.

^a^Downgraded two levels for inclusion of prospective randomized and non-randomized studies with study limitations.

#### Alignment duration and efficiency

The four RCTs [22–24, 28] reporting this outcome produced contradictory reports in relation to which bracket slot size provides faster dental arch alignment. One RCT reported a statistically significant difference (*P* < 0.05) in the number of visits required to achieve alignment between the two groups, with the 0.018-inch group achieving alignment one month faster than the 0.022-inch group [22]. Contrary to these findings, another RCT reported a significantly (*P* < 0.001) shorter time required to achieve alignment in the 0.022-inch groups with different ligation methods [23].

Two RCTs reported the overall alignment duration, comparing the difference in alignment speed between the upper and lower arches. One RCT reported no difference in alignment duration and the rate of alignment in the maxillary arch for the two slot sizes; however, alignment was significantly faster (*P* < 0.05) for the 0.022-inch group in the mandibular arch [24]. The other RCT [28] found the differences in alignment duration of both dental arches to be statistically insignificant, despite the 0.018-inch group achieving alignment 1 month before the 0.022-inch group in the upper arch.

#### Iatrogenic effects of treatment

Two RCTs [26, 27] and two NRS [31, 32] compared the amount of orthodontically induced inflammatory root resorption (OIIRR) between the two bracket systems. Two of the included studies [27, 32] assessed the severity of OIIRR using an index adapted from Malmgren *et al.* [33]. Four grades were used to quantify the amount of OIIRR, from grade 0, indicating the absence of apical root resorption, to grade 4, indicating severe apical root resorption exceeding one-third of the root length. The non-randomized prospective study [32] reported grade 0 OIIRR in 93.6% of the examined teeth for the 0.018-inch slot group, 5.6% ranged between grade 1 and 2, 0.3% ranged between grade 2 and 3, and 0.6% were grade 3. About 95.8% of the examined teeth in the 0.022-inch slot group were grade 0, with 4.2% ranging between grades 1 and 2, however, they failed to detect grade 4 severe root resorption exceeding one-third of the root length in both groups. The RCT [27] reported grade 0 in 18.9% of the 0.018-inch slot group, grade 1 in 44.6%, grade 2 in 27%, and grade 3 in 6.8% of the examined maxillary central incisors. In the 0.022-inch slot group, 29.7% had grade 0 OIIRR, 44.6% had grade 1, 14.9% had grade 2, and 8.1% had grade 3, with both groups having 2.7% grade 4 score. The other two articles [26, 31] utilized standardized periapical radiographs to measure the amount of apical root resorption in each of the comparison groups. The prevalence of OIIRR was 55% in the 0.018-inch group compared to 75% in the 0.022-inch group according to one report [26].

All articles reporting on this outcome concluded that the bracket slot size did not affect the severity of OIIRR, as no difference was found between patients treated with 0.018-inch and 0.022-inch bracket slots (*P* > 0.05).

#### Quality of orthodontic treatment outcome

Two RCTs reported on the quality of orthodontic outcomes using both bracket slot sizes. One RCT [28] utilized both the ABO cast-radiograph evaluation (CR-EVAL) and peer assessment rating (PAR) to assess this outcome. They reported a mean total ABO CR-EVAL scores of 34.7 and 34.5, and mean PAR score percentage reduction of 74.1% and 77% for the 0.018-inch and 0.022-inch groups, respectively, with no statistically significant differences between both groups for the ABO CR-EVAL and PAR score reduction (*P* > 0.05). The other RCT [23] reported on the quality of alignment utilizing the II with no statistically significant differences between the two groups (*P* > 0.05).

#### Patient-related outcomes and cost-effectiveness

Patient pain perception, in addition to OHRQoL, was explored by three of the included RCTs [25, 27, 28]. The UK study [27, 28] reported no statistically significant differences in patient pain perception after 6 months of starting the treatment, nor their perception of orthodontic treatment between the two groups (*P* > 0.05). The other RCT [25] reported a statistically significant difference between the bracket slot sizes regarding pain perception and OHRQol. The patients in the 0.022-inch group experienced less pain and had a better OHRQoL compared to the 0.018-inch group. All included studies failed to report on cost-effectiveness related to using both 0.018-inch and 0.022-inch slot sizes.

## Discussion

This systematic review comprehensively explores the effectiveness of the 0.018-inch and 0.022-inch slot sizes in patients undergoing fixed appliances treatment. Findings of this review suggest no significant statistical or clinical difference in the overall duration of fixed appliance treatment between the two slot sizes. The mean treatment duration for the 0.018-inch group ranged between 18 and 29 months; for the 0.022-inch slot group, it was between 20 and 31 months.

This variation in the overall treatment duration between the included studies might be attributed to differences in the severity of the malocclusion, visit intervals, patient cooperation, and operator-related factors. Some of the included reports were carried out at multiple sites simultaneously [28], some had numerous operators [26], while others had a single operator [30]. It has been established that the operator’s experience, operator numbers, and treatment setting affect the treatment duration [34–37]. Additionally, the malocclusions of the participants varied significantly, between almost exclusively Class I cases [30, 32], Class II [26], and more complex Class II div 1, Class II div 2, and Class III cases [28], which might influence the treatment duration [38, 39]. Another disparity is the treatment approaches presented in the included reports, as tooth extractions might increase treatment time [40].

The findings concerning the alignment duration remain contradictory. While some studies [28, 30] attributed the difference in the overall treatment duration to the working and finishing stages of the treatment, reporting no significant difference between the two slot sizes during the levelling and alignment stages [28, 30]. Others reported a statistically significant difference in the alignment duration [22–24], however, these studies had a small sample size, found it to be clinically insignificant [22], reported no difference in the alignment efficiency [23], or used single-wing brackets in the 0.018-inch group, offering less rotational control [24].

While the archwire sequence of choice can potentially impact the mean treatment duration, this appears to be primarily related to the archwire shape and cross-section, independent of the number of archwires used. Early progression into multiple rectangular archwires in the 0.018-inch slot groups might be partially responsible for the slight reduction in alignment and overall treatment times [22, 41], however, the utilization of a similar treatment protocol in both groups seems to minimize this effect.

Even though the 0.018-inch slot enables engagement of a rectangular stainless-steel archwire earlier than with the 0.022-inch slot [22, 28, 30] and indeed allows for earlier and more effective expression of torque [42, 43], this review found the claims of a significant reduction in treatment time with the 0.018-inch slot due to rapid archwire succession in the initial treatment stages to be unfounded. The conflicting reports about the efficacy of different archwire sequences and individual variations in the malocclusion make it very difficult to gauge if there is any truth to those claims. Nonetheless, if the slot size does influence the treatment time, it would be more evident in specific circumstances with extraction cases [30] and individuals with Class I malocclusion [44], where clinicians progress early to a fully engaged rectangular archwire [41]. Moreover, the manufacturing tolerances of the bracket slots further complicate the overall situation. Cash *et al.* determined that all bracket slots are oversized compared to manufacturer specifications, between 5% and 24%, which could impact the results [45].

All included studies failed to detect a significant difference in the amount of root resorption between the 0.018-inch and 0.022-inch slot sizes. It has been suggested that a narrower 0.018-inch slot might offer less clearance between the slot walls and the archwire, causing more frictional force contrary to the 0.022-inch slot; this lack of clearance and subsequent early frictional forces were thought to produce higher forces [32]. On the other hand, large-diameter rectangular archwires used alongside 0.022-inch slot brackets are known to produce significant root movement [27]. High levels of orthodontic forces and extensive root movement have been suggested as risk factors leading to OIIRR [46]. Moreover, OIIRR is a multifactorial phenomenon with various aetiological factors. These factors can be either patient related such as type of malocclusion, general health, age, gender, root form, and teeth with a history of trauma or treatment related such as treatment duration, type of tooth movement, type of orthodontic appliances and force magnitude and application [46]. However, the results indicated that bracket slot sizes do not influence the severity of OIIRR, suggesting a plausible role of genetic predisposition and individual variations [26].

The quality of treatment outcomes was comparable in individuals treated with 0.018-inch and 0.022-inch slot brackets. The UK study [28] utilized a 0.016 × 0.022-inch archwire in the 0.018-inch slot and a 0.019 × 0.025-inch archwire in the 0.022-slot brackets; these archwires/slot size combinations offer near identical play between the bracket slot and archwire, at 11.8° and 12.8°, respectively [47]. The non-significant difference reported between the two groups in both CR-EVAL and PAR scores suggests that the similar play between the archwires and slots allowed for the consistent expression of the intended prescription, minimizing any potential individual effects of slot size on incisor inclination and occlusal outcomes. This is consistent with a retrospective report using the same archwires/slot size combination [48]. The other RCT [23] compared the II between self-ligating and conventional ligating 0.018-inch and 0.022-inch slot brackets and reported no significant difference. These findings align with previous reports [44, 49–51], indicating that the bracket design in terms of ligation method, slot size, and prescription appears to have a minimal impact on alignment efficiency and occlusal outcomes.

The two RCTs reporting pain and patient perception associated with the two bracket systems gave conflicting results. The non-significant difference noted by the UK study [27, 28] was in contrast with the other RCT [25]. Curto *et al.* [25] followed up with their patients for 7 days after the commencement of fixed appliances treatment and reported a significant increase in pain and deterioration in the OHRQol in the 0.018-inch slot group. This again might be attributed to a lack of sufficient clearance between the slot walls and the initial archwire, producing more pressure and therefore pain. On the other hand, the UK study had a 6-month follow-up, which might have helped their participants to acclimate to the pressure and discomfort produced by appliance adjustments.

Factors affecting the duration of orthodontic fixed appliances treatment are complex and interlinked. Patient-related factors such as the severity of the presenting malocclusion, cooperation, and adherence to appointments might affect the treatment duration, while factors like bracket ligation, prescription, and slot size seem to have minimal impact. The effectiveness of different bracket slot sizes most likely depends on how they are combined with other variables. Case and archwire selection, along with consideration of the patient's specific needs and treatment objectives, might help optimize the advantages of specific bracket systems, enhancing treatment outcomes and potentially reducing treatment time. Therefore, the quality of treatment, side effects, and duration mostly lie within the hands of the orthodontist.

## Limitations

Despite the exclusion of multiple retrospective studies, most included RCTs and NRS were deemed to have an unclear risk of bias. The main concern was excessive dropout rates, which introduced missing data bias. This might be attributed to the lengthy and multicentre nature of some of the included studies; however, the exploratory subgroup analysis revealed that high risk of bias studies had no impact on the overall effect measure. Moreover, while the I² metric suggested no significant statistical heterogeneity, the small sample of studies eligible for inclusion in the quantitative synthesis might have influenced the quality of the overall estimates. Clinical heterogeneity was also observed across the included studies, arising from variations in patient characteristics, bracket prescriptions, treating clinician, ligation methods, bracket designs, and archwire sequences used during alignment and space closure. Despite the exclusion of patients with functional appliances treatment from most studies, the broad age range of the included sample added to the existing clinical heterogeneity. Interestingly, three [24, 26, 31] of the only four studies which included participants younger than 12 years of age reported on OIIRR. This can be attributed to the fact that at that age, all the permanent anterior teeth are usually present, which allows for the observation of OIIRR in these teeth. Additional methodological inconsistencies included variable follow-up periods and the use of different measurement units for comparable outcomes. Future researchers are encouraged to control for potential dropouts during sample size calculation and plan an intention-to-treat analysis. Furthermore, a consistent standardized clinical protocol should be established to minimize clinical heterogeneity. Future systematic reviews should attempt to include regression analyses to identify sources influencing treatment outcomes when enough studies are available. Finally, additional RCTs in this area should be rigorously designed during study inception and should provide comprehensive reporting on both clinically meaningful and patient-centred outcomes. In addition, evaluation of the cost-effectiveness of utilizing a specific bracket system or appliance should be included to better inform clinicians regarding financial implications and cost optimization.

## Conclusions

Within the limitations of this systematic review, there is some limited low-quality evidence indicating that bracket slot size does not significantly impact orthodontic treatment in terms of duration, treatment outcomes, or patient-centred variables.

## Supplementary Material

cjaf082_Supplementary_Data

## Data Availability

Data available on reasonable request.
