# Supplementary material for: Duration of fixed appliance treatment using 0.018-inch slot versus 0.022-inch slot brackets: a systematic review and meta-analysis
Source: Eur J Orthod. 2025 Oct 14;47(5):cjaf082. doi: 10.1093/ejo/cjaf082 (PMC12525148; doi:10.1093/ejo/cjaf082)
Supplement: cjaf082_Supplementary_Data [file cjaf082_supplementary_data.pdf]

Supplementary Table 1. Detailed search strategies and methods

Source	Search strategy used	Hits
**MEDLINE searched via Ovid (1946 - May 2025)**	1. Orthodontic Appliances/ OR Orthodontic Brackets/<br>2. Orthodontic Appliance Design/<br>3. ("Slot size" OR "bracket slot" OR slot* OR "edgewise bracket*" OR bracket* OR "fixed appliance*")<br>4. ("0.018" OR "0,018")<br>5. ("0.022" OR "0,022")<br>6. 1 OR 2 OR 3<br>7. 4 AND 5 AND 6	184
**PubMed (all years to May 2025)**	("Orthodontic Appliances"[Mesh] OR "Orthodontic Brackets"[Mesh] OR "Orthodontic Appliance Design"[Mesh]) OR ("Slot size" OR "bracket slot" OR slot* OR "edgewise bracket*" OR bracket* OR "fixed appliance*") AND ((“0.022 inch” OR 0,022-inch OR 0.022-inch OR 0.022) AND (“0.018 inch” OR 0,018-inch OR 0.018-inch OR 0.018))	154
**Cochrane Central Register of Controlled Trials searched via the Cochrane Library (1974 - May 2025)**	1. MeSH descriptor: [Orthodontic Brackets]<br>2. MeSH descriptor: [Orthodontic Appliances]<br>3. MeSH descriptor: [Orthodontic Appliance Design]<br>4. ("Slot size" OR "bracket slot" OR "slot") OR (edgewise NEXT bracket*) OR "bracket" OR (fixed NEXT appliance*)<br>5. (“0.018 inch” OR "0,018-inch" OR "0.018-inch" OR 0.018 OR 0,018)<br>6. (“0.022 inch” OR "0,022-inch" OR "0.022-inch" OR 0.022 OR 0,022)<br>7. (#1 OR #2 OR #3 OR #4) AND #5 AND #6	36
**Web of Science (all years to May 2025)**	TOPIC: (("Slot size" OR "bracket slot" OR slot* OR "edgewise bracket*" OR bracket* OR "fixed appliance*" OR "orthodontic brackets" OR "orthodontic appliances")) AND TOPIC: (("0.018 inch" OR 0,018-inch OR 0.018-inch OR 0.018)) AND TOPIC: (("0.022 inch" OR 0,022-inch OR 0.022-inch OR 0.022))	112

**EMBASE via Ovid (1974 - May 2025)**	1. Orthodontic Appliances/ OR Orthodontic Brackets/<br>2. Orthodontic Appliance Design/<br>3. ("Slot size" OR "bracket slot" or slot* OR "edgewise bracket*" OR bracket* OR "fixed appliance*")<br>4. ("0.018" OR "0,018")<br>5. ("0.022" OR "0,022")<br>6. 1 OR 2 OR 3<br>7. 4 AND 5 AND 6	114
**Scopus (all years to May 2025)**	(TITLE-ABS-KEY ("Slot size" OR "bracket slot" OR slot* OR "edgewise bracket*" OR bracket* OR "fixed appliance*" OR "orthodontic brackets" OR "orthodontic appliances") AND TITLE-ABS-KEY ("0.018 inch" OR 0,018-inch OR 0.018-inch OR 0.018) AND TITLE-ABS-KEY ("0.022 inch" OR 0,022-inch OR 0.022-inch OR 0.022))	63
**Manual searches**	Reference lists of potentially included studies	7
**Other bibliographic databases (all years to May 2025)**	ProQuest Dissertation and Thesis database<br>Grey literature (opengrey)<br>ISRCTN registry<br>ClinicalTrials.gov	10
**Total**		680

Supplementary Table 2. Excluded search hits based on full-text evaluation

Study	Reason for exclusion
Čelar AG, Onodera K, Bertl MH, Astl E, Bantleon HP, Sato S, Mitteroecker P. Geometric morphometric evaluations of a randomized prospective split-mouth study on modes of ligation and reverse-curve mechanics. Orthodontics & Craniofacial Research. 2014 Aug;17(3):158-69.	Outcomes of interest are not presented
Detterline DA, Isikbay SC, Brizendine EJ, Kula KS. Clinical outcomes of 0.018-inch and 0.022-inch bracket slot using the ABO objective grading system. Angle Orthodontist. 2010 May 1;80(3):528-32.	Retrospective study
El-Angbawi AM, Bearn DR, McIntyre GT. Comparing the effectiveness of the 0.018-inch versus the 0.022-inch bracket slot system in orthodontic treatment: study protocol for a randomized controlled trial. Trials. 2014 Dec;15:1-8.	Protocol
Masaes MM, Burhan AS, Youssef M, Nawaya FR. T-loop spring vs ricketts maxillary canine retractor in canine retraction efficacy and anchorage loss control: a cone-beam computed tomography study. AJO-DO Clinical Companion. 2022 Feb 1;2(1):26-40.	Retrospective study
Abdelaziz O. Evaluation of the Rate of Maxillary En-masse Retraction Using 0.018-inch Versus 0.022-inch Slot Brackets in Adults. NCT04468295, 2020.	Protocol
Nahidh M. Canine Retraction Using Different Bracket Slots' Sizes. NCT05361863, 2022.	Protocol
Soboku T, Motegi E, Sueishi K. Effect of Different Bracket Prescriptions on Orthodontic Treatment Outcomes Measured by Three-dimensional Scanning. The Bulletin of Tokyo Dental College. 2018;60(2):69-80.	Retrospective study
Thompson TL. Amount of Root Shortening Related to Type of Fixed Appliance: A Comparison of the 0.018" and 0.022" Bracket Slot. Loma Linda University, 1986.	Retrospective study
Vu CQ, Roberts WE, Hartsfield Jr JK, Ofner S. Treatment complexity index for assessing the relationship of treatment duration and outcomes in a graduate orthodontics clinic. American Journal of Orthodontics and Dentofacial Orthopedics. 2008 Jan 1;133(1):9-e1.	Retrospective study
